# Effects of Acupuncture on Oxidative Stress Amelioration via Nrf2/ARE-Related Pathways in Alzheimer and Parkinson Diseases

**DOI:** 10.1155/2021/6624976

**Published:** 2021-04-26

**Authors:** Teng-I Huang, Ching-Liang Hsieh

**Affiliations:** ^1^Department of Chinese Medicine, China Medical University Hospital, Taichung 40447, Taiwan; ^2^Chinese Medicine Research Center, China Medical University, Taichung 40402, Taiwan; ^3^Graduate Institute of Acupuncture Science, College of Chinese Medicine, China Medical University, Taichung 40402, Taiwan

## Abstract

Oxidative stress is responsible for the pathogeneses of various diseases. Mitochondrial dysfunction, impaired DNA repair, and cellular damage followed by oxidative stress contribute to neurodegenerative diseases, such as Alzheimer disease (AD) and Parkinson disease (PD). Acupuncture is a traditional therapy that has been practiced for >3000 years in Asia. Many studies have demonstrated that acupuncture has notable antioxidative, anti-inflammatory, and antiapoptotic effects. However, the exact mechanism remains unclear. Nuclear factor erythroid 2-related factor (Nrf2) is crucial in regulating the redox equilibrium. Activated Nfr2 translocates into the nucleus, binds to the antioxidant response element (ARE), and initiates antioxidative enzyme transcription. In this review, we demonstrated the effects of acupuncture on oxidative stress amelioration in AD and PD animal models through Nrf2/ARE pathway activation and Nrf2/ARE-related pathway regulation. Thus, acupuncture could be a therapeutic option for AD and PD.

## 1. Introduction

Oxidative stress is defined as the imbalance between reactive oxygen species (ROS)/reactive nitrogen species (RNS) and antioxidant defense system [1]. ROS, such as hydrogen peroxide (H_2_O_2_), superoxide radical (O_2_^•−^), and hydroxyl radical (^•^OH) [2], have a single unpaired electron in their outermost shell [3]. O_2_^•−^ can react with nitric oxide to generate peroxynitrite, a highly active RNS, and cause significant damage to intracellular components [4]. The highly reactive ROS/RNS readily interact with biomolecules to initiate a cascade of events, leading to mitochondrial dysfunction, impaired DNA repair, and cellular damage [5]—eventually contributing to the pathogeneses of neurodegenerative diseases [6].

Antioxidant enzymes, such as heme oxygenase (HO) 1), superoxide dismutase (SOD), glutathione peroxidase (GSH-Px), catalase (CAT), glutathione-*S*-transferase, and nicotinamide adenine dinucleotide phosphate (NAD(P)H) dehydrogenase [quinone] 1 (NQO1), can quench excess free radicals or facilitate a combination of free radicals with other molecules [7]. The endogenous antioxidant defense system is mainly regulated by nuclear factor erythroid 2-related factor 2 (Nrf2), one of the most important transcription factors in regulating redox equilibrium. Nrf2 can enhance the expression of downstream antioxidant genes by binding to the antioxidant response element (ARE) [8]. In addition to having an antioxidative effect, Nrf2 can modulate mitochondrial metabolic functions and protect cells from apoptosis caused by oxidative stress [9].

The brain, a complex organ containing >100 billion neurons, is susceptible to oxidative stress owing to its high oxygen consumption and lipid-rich content [10]. Neurodegenerative diseases, including Alzheimer disease (AD), Parkinson disease (PD), and Huntington disease, are caused by progressive neuron degeneration. The pathophysiologies of these diseases are complicated and warrant further clarification. Nevertheless, genetic and environmental factors are considered to be strongly associated with neurodegenerative disease initiation [11].

Acupuncture, based on the meridian theory of traditional Chinese medicine, has been practiced for >3000 years in Asia. Over time, acupuncture has evolved into several types, including manual acupuncture, electroacupuncture (EA), and laser acupuncture (LA). In manual acupuncture, medical professionals use thin and sterile metal needles to penetrate stimulation points (i.e., acupoints) on the body and manipulate the needle to achieve a state of “de-qi.” EA combines electric stimulation and acupuncture to improve the effectiveness of acupuncture. LA, a novel form of acupuncture, uses low-intensity laser to stimulate acupoints. Many studies have demonstrated the antioxidative, anti-inflammatory, and anti-apoptotic effects of acupuncture in treating diseases [12]. However, few reviews have focused on the relationship between the effects of acupuncture and redox biology. Herein, we provide an overview of several pathways in oxidative stress regulation related to acupuncture in AD and PD.

## 2. Materials and Methods

**Figure 1:**
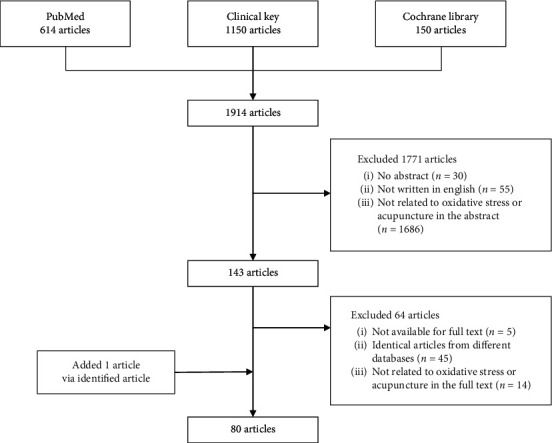
Flow of the search process.

We searched the PubMed, ClinicalKey, and Cochrane Library databases for eligible studies published from database inception to June 2020 by using Medical Subject Headings keywords alone or in combination: “acupuncture,” “Alzheimer's disease,” “Parkinson's disease,” “oxidative damage,” “oxidative stress,” and “Nrf2.” The search was limited to only English-language articles. First, the search yielded 1914 articles. After manual screening, we excluded 1771 articles including those without abstracts or with abstracts lacking the terms “oxidative stress” or “acupuncture” and obtained 143 articles for further screening. Next, we excluded 64 articles, including those with unavailable full text, those identical to articles from other databases, or those lacking the terms “oxidative stress” or “acupuncture” in the main text; we also added one article identified from the references cited in a selected article. Finally, 80 articles were included in the review. The flow of the search process is shown in Figure 1.

## 3. Results and Discussion

### 3.1. Nrf2/ARE-Related Signaling Pathways in Oxidative Stress

Nrf2, belonging to the leucine zipper transcription factor family, is a crucial transcription factor in regulating redox equilibrium. Nrf2 consists of seven functional domains, spanning from Nrf2-ECH homology (Neh) 1 to Neh7 : Neh1 allows Nrf2 binding to DNA in the nucleus; Neh2 promotes Nrf2 binding to kelch-like ECH-associated protein 1 (Keap1, which is responsible for Nrf2 ubiquitination and degradation in cytosol) [13, 14]; Neh3–Neh5 are associated with the activation of ARE genes and transcriptional coactivators; Neh6 is a serine-rich region regulated by glycogen synthase kinase (GSK) 3*β*, which can phosphorylate specific residues in Neh6 and thus induce nuclear export and proteasome degradation functions of Nrf2 [15]; and finally, Neh7 binds to the retinoid acid receptor alpha and represses Nrf2 activity. Under homeostatic conditions, Nrf2 is predominantly present in the cytosol and binds to Keap1. Keap1 acts as an adaptor protein for cullin-3/RING box protein 1-dependent E3 ubiquitin ligase complex and further promotes ubiquitination and degradation of Nrf2 [16]. On exposure to oxidative stress, the Keap1 structure changes, allowing Nrf2 to detach from Keap1 and translocate into the nucleus. After translocation into the nucleus, Nrf2 binds to ARE and enhances downstream antioxidant gene expression to maintain the redox balance in cells [17]. The Nrf2 nuclear translocation and ARE transactivation processes are regulated by multiple pathways including protein kinase C (PKC), phosphatidylinositol-3-kinase (PI3K)/protein kinase B (Akt)/GSK-3*β*, p38 mitogen-activated protein kinase (MAPK), and nuclear factor kappa B (NF-*κ*B).

#### 3.1.1. PKC

PKC isozymes belong to the serine/threonine kinase family and are highly associated with cell apoptosis and autophagy. The alternation of PKC activities contributes to the progression of several neurodegenerative diseases [18, 19]. PKCs can phosphorylate Nrf2 at Ser40, which lies in Neh2, and further promote the dissociation of Nrf2 from Keap1 and nuclear translocation [20]. However, Nrf2 stabilization and accumulation in the nucleus are dependent on cellular Src activation, which is induced by H_2_O_2_ and regulated by PKC*δ* phosphorylation at Tyr311 [21].

### 3.2. PI3K/Akt/GSK-3*β*

The PI3K/Akt pathway, essential in cell growth, survival, differentiation, and metabolism, is necessary for neuroprotection against oxidative damage [1]. This pathway is typically activated by trophic factors such as nerve growth factor or brain-derived neurotrophic factor (BDNF) [22]. BDNF, a neurotrophin, is essential for inducing the nuclear translocation of Nrf2. In a recent study [23], incubating hippocampal neurons of rats with BDNF (50 ng/mL) for 6 h was noted to significantly enhance Nrf2 nuclear translocation. However, this effect disappeared when PI3K was blocked. These results suggest that BDNF can promote Nrf2 nuclear translocation via the PI3K pathway. Another study [24] reported that cortical and hippocampal BDNF levels decreased in Nrf2^−/−^ mice, indicating that Nrf2 is also crucial in BDNF expression regulation. Moreover, when BDNF binds to tropomyosin-related kinase receptor type B (TrkB.T1), p75 neurotrophin receptor (p75^NTR^) is not inhibited, and ceramide can be generated with p75^NTR^. At low levels, ceramide can activate PKC*ζ*, further activating casein kinase 2 [25]—which is crucial for nuclear translocation and transcription activation of Nrf2 [26].

PI3K/Akt pathway activation can cause inhibitory phosphorylation of GSK-3*β*. As a negative regulator of Nrf2, GSK-3*β* enhances nuclear export and Nrf2 degradation via both direct and indirect pathways. In the direct pathway, GSK-3*β* phosphorylates the specific residues in the Neh6 domain of Nrf2, targeting it for degradation through SCF*β*/TrCP, whereas in the indirect pathway, GSK-3*β* promotes Src kinase accumulation in the nucleus and further results in Nrf2 phosphorylation at Tyr568, leading to Nrf2 nuclear export and degradation [27, 28].

#### 3.2.1. p38 MAPK

MAPK belongs to the silk proteins/threonine kinase family. One of the most studied subpathways of MAPK is p38 MAPK, which plays a crucial role in cell growth, apoptosis, and inflammation. p38 MAPK promotes an association between Nrf2 and Keap1 and limits the nuclear accumulation of Nrf2 in the human hepatoma cell line HepG2 [29]. However, Jung et al. [30] demonstrated that p38 MAPK inhibition significantly decreases the binding of the nuclear proteins to ARE and ARE-mediated transcriptional activity in C2 ceramide-treated astrocytes cells, indicating that p38 MAPK activation is crucial for activating the ARE-related antioxidative effects. Thus, p38 MAPK has a dual characteristic in regulating neurological diseases: neurotoxicity induction in the acute phase and neuroprotective antiapoptotic effect promotion in the subacute phase [31].

#### 3.2.2. NF-*κ*B

In addition to Nrf2, NF-*κ*B is another key transcription factor regulating cellular responses to oxidative stress [32]. However, the effects of Nrf2 and NF-*κ*B are completely contradictory. Oxidative stress promotes NF-*κ*B inhibitor (I*κ*B) phosphorylation and degradation and further enhances NF-*κ*B release and nuclear translocation. After translocation into the nucleus, NF-*κ*B binds to DNA and initiates the transcription of proinflammatory cytokines, such as interleukins 1 and 6, tumor necrosis factor (TNF) *α*, and inducible nitric oxide synthase [8]. The proinflammatory molecules further enhance oxidative stress in the cell, forming a vicious cycle.

Nrf2, which exhibits a notable ability of maintaining redox equilibrium, can inhibit NF-*κ*B pathway activation in two modes: (1) reducing ROS levels by increasing the levels of transcription and releasing antioxidative enzymes and (2) reducing NF-*κ*B nuclear translocation by preventing I*κ*B degradation [33]. By contrast, NF-*κ*B can also inhibit the antioxidative effect of Nrf2 by blocking the ARE region and thus preventing ARE gene transcription [8]. In other words, NF-*κ*B and Nrf2 are redox-regulated transcription factors that can interfere with one another.

**Figure 2:**
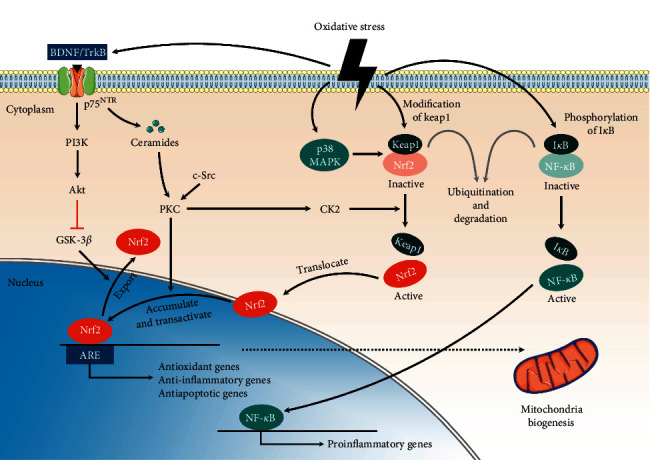
Summary of Nrf2/ARE-related pathways. Under homeostasis condition, Nrf2 and NF-*κ*B would bind to the inhibitors and further be degraded. Oxidative stress induces modification of Keap1 and phosphorylation of I*κ*B, resulting in the dissociation of Nrf2 from Keap1 and NF-*κ*B from I*κ*B. The active Nrf2 translocates into the nucleus, binds to ARE, and initiates antioxidants transcription. p38 MAPK would enhance the combination of Nrf2 and Keap1, thus reducing the active Nrf2. BDNF can enhance the accumulation and transactivation of Nrf2 via PKC pathway. GSK-3*β* would enhance Nrf2 nuclear export and degradation, which would be inhibited by PI3K/Akt pathway. Furthermore, Nrf2 can promote mitochondria biogenesis. BDNF: brain-derived neurotrophic factor; TrkB: tropomyosin-related kinase receptor type B; p75 ^NTR^: p75 neurotrophin receptor; PI3K: phosphatidylinositol-3-kinase; Akt: protein kinase B; GSK-3*β* : glycogen synthase kinase-3 beta; Nrf2: nuclear factor erythroid 2-related factor 2; ARE: antioxidant response element; PKC: protein kinase C; c-Src: cellular src; CK2: casein kinase 2; MAPK: mitogen-activated protein kinase; Keap1: kelch-like ECH-associated protein 1; I*κ*B : NF-*κ*B inhibitor; NF-*κ*B: nuclear factor kappa B.

The Nrf2/ARE-related signal transduction pathways are illustrated in Figure 2.

### 3.3. Effect of Acupuncture on Oxidative Stress Amelioration via Nrf2/ARE-Related Pathways in AD

#### 3.3.1. Oxidative Stress and AD

AD, the most common cause of dementia, is expected to affect >131 million people by 2050 [34]. The clinical manifestations of AD include progressive cognitive decline (including memory loss), behavioral change, and language impairment. The pathological characteristics of AD include extracellular aggregation of amyloid beta (A*β*) plaques, intracellular neurofibrillary tangles, and loss of cholinergic neurons and synapses [34, 35].

A*β*_1–40/42_ peptides are the product of amyloid precursor protein (APP) metabolism that are cleaved consecutively by *β*- and *γ*-secretases. In healthy brains, A*β*_1–40/42_ peptides are degraded by A*β*-degrading proteases. Nevertheless, in patients with AD, due to metal homeostasis disruption, A*β* peptides further interact with metal ions, such as zinc, copper, and iron, and form A*β* oligomers and then fibrils [36]. A*β* accumulation and metal ion misregulation both induce oxidative stress [36].

In addition to destroying protein, lipid, and DNA structures, oxidative stress increases mitochondrial dysfunction and activate downstream caspases, leading to apoptosis [37]. Under severe oxidative stress, mitochondrial permeability transition pores, which are regulated by the B-cell lymphoma 2 (Bcl-2) family [38–40], open to allow protons, Ca^2+^, and large molecules (molecular mass up to 1500 Da, such as GSH) to pass through the inner membrane of mitochondria. Proton gradient loss leads to mitochondrial depolarization, resulting in respiratory chain uncoupling, ROS hypergeneration, substantial release of matrix Ca^2+^, and depletion of GSH and other reductants [37, 41]. The increase in intracellular Ca^2+^ activates cyclin-dependent kinase 5, resulting in the hyperphosphorylation of the tau protein and self-assembly of neurofibrillary tangles [42]. Moreover, the opening of the mitochondrial permeability transition pore induces the release of cytochrome c, caspase-9, proapoptotic factors, and apoptotic protease-activating factor-1, thereby leading to apoptotic cell death [37].

#### 3.3.2. Effect of Acupuncture in AD

Acupuncture improves cognitive function by increasing the connectivity between cognition-related regions, including the insula, dorsolateral prefrontal cortex, hippocampus, thalamus, inferior parietal lobule, and anterior cingulate cortex [43, 44]. This effect is caused by regional brain blood flow increase, neurotransmitter modulation, synaptic plasticity improvement, endogenous antioxidant defense system enhancement, and neuronal apoptosis attenuation [1, 45–49]. Moreover, several studies revealed that acupuncture may induce neurogenesis [50]. In the studies we reviewed, *Zusanli* (ST36) and *Baihui* (GV20) are the most commonly used acupoints to enhance brain cell proliferation. Kim et al. [51] demonstrated that acupuncture at *Zusanli* (ST36) increased cell proliferation in the dentate gyrus of ischemic gerbils. Huang et al. [52] revealed that both acupuncture and EA at *Zusanli* (ST36) and *Baihui* (GV20) enhanced cell proliferation in the subgranular zone of the dentate gyrus. Though acupuncture appeared to enhance neurogenesis, further research exploring the mechanisms and pathways is needed.

*(1) Nrf2/ARE Pathway*. Zhou et al. [53] reported that EA at *Baihui* (GV20) enhances neurogenesis and the expression of Nrf2, HO-1, and BDNF in the hippocampus of enhanced single prolonged stress-treated rats. The neuroprotective effect of EA pretreatment would be blocked in Nrf2-knockdown models. Another study [13] revealed that acupuncture at *Baihui* (GV20) and *Zusanli* (ST36) ameliorates cognitive impairment and hippocampus neuronal loss in models of vascular dementia, accompanied by significant enhancement of HO-1 and NQO1 protein levels in the hippocampus. Interestingly, the neuroprotective effect of acupuncture is also abolished in Nrf2^(-/-)^ mice, which indicates that EA protects neuronal loss and promotes neurogenesis via the Nrf2/HO-1 pathway. A study [14] demonstrated that EA at *Zusanli* (ST36) reduced plasma TNF-*α* and interleukin-6 levels, increased SOD, GSH-Px, and CAT levels, and increased HO-1 and Nrf2 expression considerably, suggesting that EA attenuates oxidative stress-induced-tissue injury by activating the Nrf2/ARE pathway.

Several studies have reported that Nrf2 can upregulate antiapoptotic proteins Bcl-2 and Bcl-xL and thus enhance cell survival and prevent cellular apoptosis [54, 55]. A study [56] demonstrated that acupuncture at the acupoints of *Shenting* (GV24) and *Benshen* (GB13) could suppress oxidative stress by upregulating Bcl-2 expression and downregulating Bax, cytochrome c, and caspase 3 and 9 expression, thereby reducing the concentrations of ROS and malondialdehyde (MDA; a lipid peroxidation product) and increasing SOD generation. The antiapoptotic effect of acupuncture may be mediated by Nrf2. However, further research is needed to elucidate possible mechanisms.

*(2) Nrf2/ARE-Related Pathways*. In addition to activating Nrf2/ARE directly, acupuncture has shown neuroprotective and antioxidative effects via other Nrf2/ARE-related pathways, including PKC, PI3K/Akt/GSK-3*β*, p38 MAPK, and NF-*κ*B. EA increased PKC expression in the hippocampus of a rat depression model [57]. However, studies that have explored the effect of acupuncture on the PKC pathway in an AD model are limited. EA at *Baihui* (GV20) with disperse waves of 1 and 20 Hz for 30 min daily for 4 weeks can significantly improve learning and memory functions and upregulate BDNF expression levels in APP/PS1 transgenic mice [58, 59]. BDNF can promote Nrf2 nuclear translocation via the PI3K pathway, further bind to ARE, and initiate transcription of antioxidants. Yu et al. found that high-frequency (50-Hz) EA could downregulate GSK-3*β* activity and ameliorate cognitive impairment in rats [60]. Mammalian target of rapamycin (mTOR), the downstream factor of PI3K/Akt pathway, is a leading autophagy regulator [61]. A reduction of mTOR activity triggers autophagy to decrease the deposition of A*β* plaques and improve memory impairment [62]. Liu et al. found that EA at *Baihui* (GV20) could decrease mTOR levels in APP/PS1 transgenic mice [63]. In a rat AD model, EA at *Baihui* (GV20), *Taixi* (KI3), and *Zusanli* (ST36) with 1 mA, 2 Hz for 15 min daily for 12 sessions could restrain the inflammatory reaction in the CNS by reducing p38 MAPK levels [64]. A study [65] revealed that oxidative stress induces the activation of NF-*κ*B and its target gene TP53 and that acupuncture at the *Zusanli* (ST36) acupoint for 30 seconds, once daily (with a rest every seventh day) for 2 weeks, can inhibit the nuclear translocation of NF-*κ*B and TP53 expression in the hippocampus in a multi-infarct model. EA at *Baihui* (GV20), *Yintang* (EX-HN3), and *Shuigou* (GV26) could reduce *β*-secretase1 deposition, which is regulated by NF-*κ*B, in APP/PS1 transgenic mice [66].

(3) *Other Effects of Acupuncture on Oxidative Stress Amelioration*. In addition to enhancing the endogenous antioxidant defense system by activating Nfr2/ARE, acupuncture can decrease ROS generation directly by regulating NADPH oxidases (NOXs) and adenosine monophosphate-activated protein kinase (AMPK) pathway. EA at *Baihui* (GV20) and *Yongquan* (KI1) can inhibit NOX2 expression and further reduce the hippocampal accumulation of MDA and 8-hydroxy-2′-deoxyguanosine (a DNA damage biomarker) [67]. Peroxisome proliferator-activated receptor gamma coactivator 1-alpha (PGC-1*α*), regulated by AMPK, can decrease ROS generation by regulating mitochondrial biogenesis and degrading damaged mitochondria through the autophagy–lysosome machinery [68]. EA upregulated PGC-1*α* expression and also improved energy metabolism in the brains of senescence accelerated mouse-prone 8 mice [69].

The heat shock protein (Hsp) family can protect cells against apoptosis under stress by degrading misfolded proteins and preventing denatured protein aggregation [70]. Chang et al. noted that acupuncture at *Danzhong* (CV17), *Zhongwan* (CV12), *Qihai* (CV6), bilateral *Xuehai* (SP10), and *Zusanli* (ST36) could reduce oxidative protein damage and promote Hsp84 and Hsp86 expression, which may delay brain aging and prevent neurodegeneration [70].

**Figure 3:**
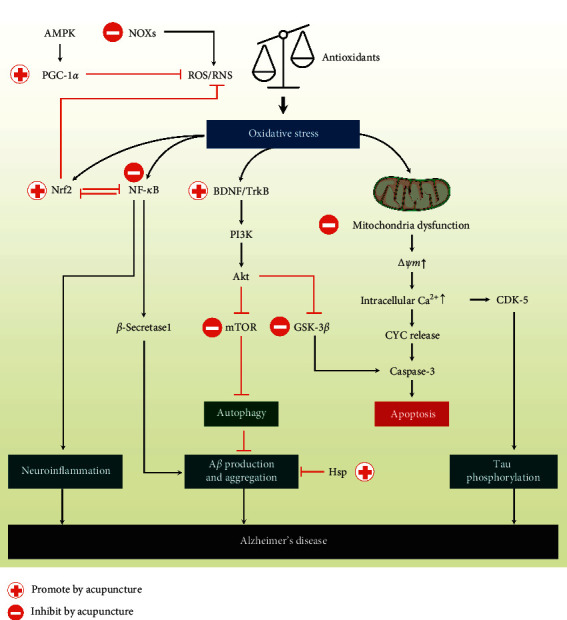
Summary of effect of acupuncture on oxidative stress amelioration in AD. Except for Nrf2/ARE-related pathways, acupuncture can ameliorate oxidative stress and decrease neuroinflammation, A*β* production, and aggregation, and tau phosphorylation via multiple signal transduction pathways. Acupuncture can reduce the generation of ROS by activating Nrf2 and PGC-1*α*, and inhibiting NOXs. Moreover, acupuncture can reverse mitochondria dysfunction, further reducing tau phosphorylation. ROS: reactive oxygen species; RNS: reactive nitrogen species; AMPK: adenosine monophosphate-activated protein kinase; PGC-1*α*: peroxisome proliferator-activated receptor gamma coactivator 1-alpha; NOX: nicotinamide adenine dinucleotide phosphate oxidases; Nrf2: nuclear factor erythroid 2-related factor 2; NF-*κ*B: nuclear factor kappa B; BDNF: brain-derived neurotrophic factor; TrkB: tropomyosin-related kinase receptor type B; PI3K: phosphatidylinositol-3-kinase; Akt: protein kinase B; mTOR: mammalian target of rapamycin; A*β*: amyloid beta; GSK-3*β*: glycogen synthase kinase-3 beta; Hsp: heat shock protein; ∆*ψ*m: the mitochondrial membrane potential; CYC: cytochrome C; CDK-5: cyclin-dependent kinase 5.

In summary, acupuncture ameliorates oxidative stress in AD in five ways: (1) reduction of oxidative stress (increasing antioxidant generation and decreasing ROS generation), (2) suppression of apoptosis (regulating signaling pathways downstream of ROS to suppress cell apoptosis), (3) reduction of A*β* production and deposition, (4) repair of ROS-damaged proteins, lipids, and DNA, and (5) neuroinflammation relief. Effects of acupuncture on oxidative stress amelioration in AD are demonstrated in Figure 3.

### 3.4. Effect of Acupuncture on Oxidative Stress Amelioration via Nrf2/ARE-Related Pathways in PD

#### 3.4.1. Oxidative Stress and PD

PD, a progressive neurodegenerative disease, is characterized by motor (bradykinesia, resting tremor, rigidity, and postural instability) and nonmotor (cognitive impairment, psychiatric symptoms, and dysautonomia) symptoms [71, 72]. PD pathology includes Lewy body formation and dopaminergic neuron degeneration in the substantia nigra [1]. The PD etiology remains unclear; however, PD is strongly associated with oxidative stress. Several studies have suggested that oxidative stress contributes to the formation of Lewy bodies and the degeneration of dopamine cells in PD [73]. Iron (Fe) metabolism disruption, GSH depletion, and MDA (a lipid oxidation biomarker) elevation have been observed in patients with PD [1]. The equilibrium between apoptosis and autophagy is crucial in the degradation of *α*-synuclein, the major Lewy body component [74].

#### 3.4.2. Effect of Acupuncture on PD

Acupuncture can improve motor and nonmotor symptoms in patients with PD and animal models [75] by reducing oxidative stress, modulating neurotransmitters, and attenuating neuronal loss [76–78].

(1) *Nrf2/ARE Pathway*. In 6-hydroxydopamine (6-OHDA) rats, acupuncture at *Yanglingquan* (GB34), *Taichong* (LV3), *Zusanli* (ST36), and *Xuehai* (SP10) acupoints increased SOD and GSH-Px levels, reduced MDA level, and inhibited oxidative stress [79]. Moreover, in the striatum of 1-methyl-4-phenyl-1,2,3,6-tetrahydropyridine- (MPTP-) treated mice, acupuncture at *Yanglingquan* (GB34) could enhance SOD and CAT activities [80]. High-frequency (100-Hz) EA at *Zusanli* (ST36) and *Sanyinjiao* (SP6) acupoints can reduce H_2_O_2_ and MDA levels and increase SOD, GSH, and GSH-Px levels [81]. Wattanathorn et al. found that LA at *Shenmen* (HT7) reduced MDA and monoamine oxidase type B levels and increased GSH-Px levels in the hippocampus of 6-OHDA rats [82].

Nrf2-deficient mice are hypersensitive to PD-generating neurotoxins [83]. A study [84] revealed that high-frequency (100-Hz) EA at *Zusanli* (ST36) and *Sanyinjiao* (SP6) could reverse the suppression of the Nrf2/ARE system induced by MPTP in a mouse model of PD, indicating that EA exhibits notable antioxidative effects by increasing Nrf2/ARE expression. A similar upregulating effect of EA on Nrf2/ARE expression was reported in another animal study [85].

(2) *Nrf2/ARE-Related Pathways*. Acupuncture at *Yanglingquan* (GB34) can protect dopaminergic neurons from apoptosis and improve motor symptoms by triggering the PI3K/Akt cascade [86]. In an animal model of PD, acupuncture at *Yanglingquan* (GB34) promoted the autophagic clearance of *α*‐synuclein by activating an mTOR-independent pathway [87], which is positively regulated with PI3K/Akt cascade.

In the MPTP mouse model, EA at *Baihui* (GV20) and *Yintang* (GV29) could increase BDNF expression [88]. Notably, Liang et al. compared the effect of different frequencies of EA (0, 2, and 100 Hz) on BDNF regulation in the substantia nigra and ventral tegmental area of PD mice and found that only high-frequency (100-Hz) EA significantly increased the BDNF level [89].

Several studies have reported that EA can protect cells from oxidative-stress-induced injury by inhibiting p38 MAPK pathway [90–93]. In acute-phase oxidative stress-induced injury, p38 MAPK enhances the link between Nrf2 and Keap1 and decreases Nrf2 nuclear accumulation. Thus, p38 MAPK inhibition could aid translocation and accumulation of Nrf2 in the nucleus. Nevertheless, few studies have explored the effect of acupuncture on regulating PD and p38 MAPK.

Acupuncture attenuates cognitive impairment and oxidative stress by reducing NF-*κ*B expression in the rat cerebral multi-infarct model [65]. NF-*κ*B inhibits TP53, which is highly associated with apoptosis [94]. Park et al. revealed that acupuncture showed predominant neuroprotective effects in the rat PD model. However, these protective effects were abrogated in the p53-knockout mice, indicating that p53 mediates acupuncture-induced neuroprotection in PD [95].

In summary, acupuncture lowers oxidative stress in PD in three ways: (1) reduction of oxidative stress (increasing antioxidant generation and decreasing ROS generation), (2) suppression of apoptosis (regulating signaling downstream of the ROS pathway to suppress cell apoptosis), and (3) reduction of *α*‐synuclein production and deposition.

**Figure 4:**
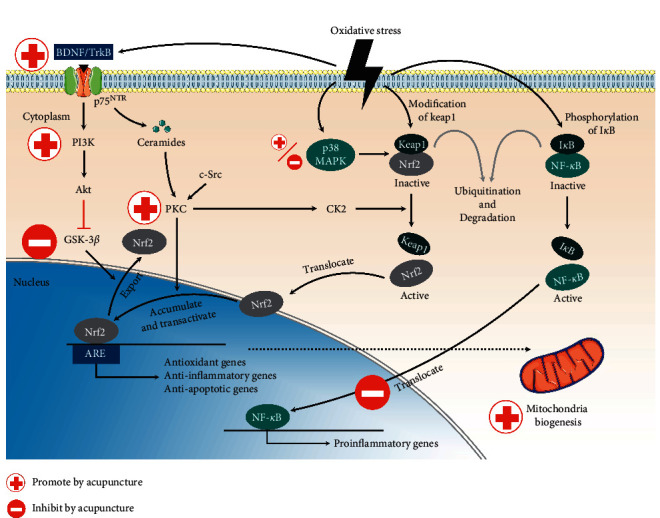
Summary of effect of acupuncture on oxidative stress amelioration via Nrf2/ARE-related pathways. Acupuncture can upregulate the expression of BDNF, PI3K/Akt, and PKC and inhibit GSK-3*β* pathway, further increasing the nuclear translocation, accumulation, and transactivation of Nrf2. In addition, acupuncture can inhibit NF-*κ*B translocate into the nucleus, hence downregulating the expression of proinflammatory genes. Moreover, acupuncture can promote mitochondria biogenesis. Interestingly, effect of acupuncture on p38 MAPK pathway has a dual characteristic. BDNF: brain-derived neurotrophic factor; TrkB: tropomyosin-related kinase receptor type B; p75 ^NTR^: p75 neurotrophin receptor; PI3K: phosphatidylinositol-3-kinase; Akt: protein kinase B; GSK-3*β* : glycogen synthase kinase-3 beta; Nrf2: nuclear factor erythroid 2-related factor 2; ARE: antioxidant response element; PKC: protein kinase C; c-Src: cellular src; CK2: casein kinase 2; MAPK: mitogen-activated protein kinase; Keap1: kelch-like ECH-associated protein 1; I*κ*B : NF-*κ*B inhibitor; NF-*κ*B: nuclear factor kappa B.

Effects of acupuncture on ameliorating oxidative stress via Nrf2/ARE-related pathways in AD and PD are illustrated in Figure 4.

## 4. Conclusions

Acupuncture exhibits notable neuroprotective and antioxidative effects by activating the Nrf2/ARE pathway. The nuclear translocation and accumulation of Nrf2 are regulated via several pathways including PKC, PI3K/Akt/GSK-3*β*, p38 MAPK, and NF-*κ*B. In addition to increasing the activity of endogenous antioxidants, acupuncture reduces ROS generation and inhibits neuronal apoptosis. Moreover, acupuncture can promote the repair of ROS-damaged lipids, proteins, and DNA and improve autophagy, thus reducing the production or deposition of pathological products, such as A*β* and Lewy bodies. This review article demonstrated that acupuncture effectively ameliorates oxidative stress in AD and PD animal models by activating the Nrf2/ARE pathway and regulating the Nrf2/ARE-related pathways. Thus, acupuncture could be a therapeutic option for AD and PD. However, acupuncture has demonstrated dual-regulatory characteristics in several pathways, such as p38 MAPK and p53. Therefore, further research focusing on the molecular mechanisms of acupuncture is needed.

## Data Availability

The data used to support the study are available from the corresponding author upon request.
